# Varicella in Pregnancy: Five Priorities for Clinicians

**DOI:** 10.1155/S1064744994000013

**Published:** 1994

**Authors:** Patrick Duff

**Affiliations:** Division of Maternal-Fetal Medicine University of Florida College of Medicine Gainesville Florida USA


Infectious Diseases in Obstetrics and Gynecology 1:163–165 (1994)
© 1994 Wiley-Liss, Inc.

### Varicella in Pregnancy: Five Priorities for Clinicians

Acute varicella (chicken pox) is one of the most highly contagious viral illnesses. The infection occurs commonly in childhood, and approximately 95% of individuals are immune by the time they reach childbearing age. Varicella occurs in fewer than 1:1,000 pregnancies each year.[1] Although it is a relatively mild illness in children, varicella may cause severe complications in pregnant women and their fetuses. Accordingly, physicians and nurses who provide obstetric care must be aware of the following 5 priorities.

First, at the time of the patient's first prenatal appointment, she should be questioned about a history of varicella. If she is certain she had chicken pox, she should be reassured that second infections are extremely unlikely and that she need not fear accidental exposure to a person with varicella. If the patient is uncertain about her history and a family member is unable to verify that infection occurred, a serologic test to determine immune status should be performed.[2] This test is particularly important in women who have frequent occupational exposure to children with viral illnesses, e.g., school teachers, day care workers, and health care workers. The appropriate serologic test for determining immune status is measurement of IgG antibody by an enzyme-linked immunosorbent assay (ELISA) or the fluorescent antibody test for membrane antigen (FAMA). Approximately 80% of patients with uncertain histories will, in fact, have evidence of immunity, and they can be reassured that they are not at risk during the present pregnancy. Susceptible women must be specifically counseled to avoid exposure to other individuals who may have varicella.

The second priority is to promptly and correctly evaluate the obstetric patient who has been exposed to an individual with acute varicella and whose serologic status is either unknown or negative. In the former case a serologic test should immediately be performed. Laboratory personnel must be informed of the need for completion of the test within 24–48 h. If the patient is seronegative or if the laboratory test cannot be completed promptly, the patient should be offered varicella-zoster immune globulin (VZIG). This preparation contains high titer of antibody to the varicella-zoster virus, and, if it is given within 4 days of exposure, is of benefit in preventing or attenuating subsequent illness. The appropriate dose of VZIG is 1 vial (125 units) per 10 kg body weight, administered intramuscularly, up to a maximum of 5 vials (625 units).[3] The approximate cost of VZIG is $400–$600.

The third priority is to treat appropriately patients who present for care more than 4 days after exposure or who become infected despite receiving VZIG. These patients should be counseled about the major complications of varicella: disseminated infection, which is extremely uncommon in immunocompetent patients; encephalitis, which occurs in $\leq 1\%$ of patients; and pneumonia, which may occur in 20–50% of patients. In reports published prior to the availability of effective antiviral chemotherapy, the mortality in pregnant women with varicella pneumonia was as high as 40%.[4] Patients should be instructed to report immediately if signs of severe systemic illness develop. If evaluation confirms the presence of a serious complication, the patient should be hospitalized and treated with intravenous acyclovir, 10–15 mg/kg or 500 mg/$m^2$ 3 times a day for approximately 7 days.[4] Consultation with specialists in infectious diseases, pulmonology, and critical care may be indicated.

The fourth priority is to evaluate exposed fetuses for possible congenital varicella infection. Recent reports have shown that the incidence of anatomic anomalies related to maternal varicella infection ranges from 0 to 9%.[1,5] Serologic evidence of congenital infection may be present in up to 25% of exposed neonates. Several diagnostic tests have been proposed for identification of affected fetuses, including amniocentesis, chorionic villus sampling (CVS), cordocentesis, and ultrasound.[6,7] Identification of virus, viral antigen, or antibody in amniotic fluid, placental tissue, or fetal blood confirms that infection has occurred but does not indicate that the fetus has been seriously injured. The best test for identifying the fetus with anomalies is ultrasound. Ultrasound findings suggestive of congenital varicella include, but are not limited to, polyhydramnios, hydrops, ventriculomegaly, microcephaly, cardiac anomalies, limb abnormalities, intrauterine growth retardation, and dystrophic calcifications in multiple organs, especially the liver.[7] Mothers with affected infants may be offered pregnancy termination if the gestational age is appropriate.

The fifth priority is to provide appropriate care to the mother and neonate when the former is acutely infected at the time of delivery. When infection occurs during the period 5 days before to 2 days after delivery, the fetus may be exposed to an intense viremia without benefit of transplacental transfer of maternal antibody. Up to 40% of these infants develop neonatal varicella, and the mortality in untreated infants approaches 20%.[8] Accordingly, the pediatrician who will be caring for the infant must be notified promptly that an acutely infected mother is about to deliver. Immediately following delivery, the infant should receive VZIG at the direction of the pediatrician.[8] In addition, mother and infant should be separated at least until neonatal assessment has been completed and immunoprophylaxis has been administered.

In summary, varicella in pregnancy is a rare, but potentially fatal, disorder for both mother and fetus. The immune status of patients should be determined early in pregnancy. Susceptible patients are candidates for immunoprophylaxis if exposure occurs. Infected patients should be monitored for serious complications, and their fetuses should be assessed for congenital infection with ultrasound examination. Infants at risk for neonatal varicella are also candidates for immunoprophylaxis with VZIG.

*Patrick Duff, M.D.*
*Division of Maternal-Fetal Medicine*
*University of Florida College of Medicine*
*Gainesville, Florida*

### REFERENCES

1. Balducci J, Rodis JF, Rosengren S, Vintzilleos AM, Spivey G, Vosseller C: Pregnancy outcome following first-trimester varicella infection. Obstet Gynecol 79:5–6, 1992.
2. McGregor JA, Mark S, Crawford GP, Levin MJ: Varicella zoster antibody testing in the care of pregnant women exposed to varicella. Am J Obstet Gynecol 157:281–284, 1987.
3. Varicella-zoster immune globulin for the prevention of chickenpox. Morbidity and Mortality Weekly Report 33:83–100, 1984.
4. Smego RA, Asperilla MO: Use of acyclovir for varicella pneumonia during pregnancy. Obstet Gynecol 78:1112–1116, 1991.

5. Paryani SG, Arvin AM: Intrauterine infection with varicella-zoster virus after maternal varicella. N Engl J Med 314:1542–1546, 1986.
6. Grose C, Itani O, Weiner CP: Prenatal diagnosis of fetal infection: Advances from amniocentesis to cordocentesis—congenital toxoplasmosis, rubella, cytomegalovirus, varicella virus, parvovirus and human immunodeficiency virus. Pediatr Infect Dis 8:459–468, 1989.
7. Pretorius DH, Hayward I, Jones KL, Stamm E: Sonographic evaluation of pregnancies with maternal varicella infection. J Ultrasound Med 11:459–463, 1992.
8. Brunell PA: Varicella in pregnancy, the fetus, and the newborn: Problems in management. J Infect Dis 166(Suppl 1):S412–S417, 1992.
